# Editorial: Genetic diversity and selection signatures in composite breeds

**DOI:** 10.3389/fgene.2022.992609

**Published:** 2022-08-17

**Authors:** Tiago do Prado Paim, El Hamidi Hay, Luiz F. Brito

**Affiliations:** ^1^ Instituto Federal Goiano, Rio Verde, GO, Brazil; ^2^ USDA Agricultural Research Service, Fort Keogh Livestock and Range Research Laboratory, Miles City, MT, United States; ^3^ Department of Animal Sciences, Purdue University, West Lafayette, IN, United States

**Keywords:** ancestry, hair sheep, genomic breed composition, genetic resources, environmental adaptability, genetic structure

Since domestication of livestock species, numerous breeds have been developed through natural and artificial selection for specific traits. In addition to organized crossbreeding schemes (e.g., in poultry and swine breeding industries), composite breeds have also been created through crossing breeds with complementary characteristics for multiple generations. As composite breeds also undergo intensive selection, signatures of selection could be formed in their genome, which could provide important background knowledge on their selection history and genomic structure. However, most previous studies focused on pure breeds. Therefore, this Research Topic was designed to compile original studies investigating the genetic diversity of composite breeds and potential footprints of selection in their genome and demonstrate the usefulness of genomic information to understand population structure, breed formation, and better understand the genetic background of local genetic resources. This Research Topic includes 15 papers, in which three of them investigated composite breeds of beef cattle (Crum et al.; Mulim et al.; Vahedi et al.), eight papers focused on Chinese genetic resources, including cattle (Zhang et al.; Jin et al.; Liu et al.; Luo et al.; Sun et al.), pigs (Xu et al.; Yang et al.), and sheep (Guo et al.). The other papers focused on Korean synthetic pigs (Kim et al.), Copy Number Variation (CNV) in chicken (Chen et al.), and hybrid ass (Dong et al.). Lastly, Qiao et al. reported a new hair sheep reference genome based on the Dorper breed, which originated in South Africa through crossing of Dorset Horn and Blackhead Persian sheep.

All composite cattle breeds studied here were indicine (*Bos taurus indicus*) × taurine (*Bos taurus*) crossbred. Crum et al. identified taurine and indicine haplotype representation in three American Composite cattle breeds (Brangus, Beefmaster, Santa Gertrudis). Lower than expected levels of Brahman contribution were found across the genome of the composite breeds. The average Brahman genome content was 25.81 ± 8.01% (±standard deviation among sampled individuals) for Brangus; 27.60 ± 7.05% for Santa Gertrudis; and 30.84 ± 7.48% for Beefmaster. These authors found strong evidence that selection for polledness, coat color, growth, calving ease, and intra-muscular fat content produced early-generation cattle with lower than expected indicine proportion in the genomes of all three breeds. Vahedi et al. studied the same three composite breeds using a different dataset and different analytical methods. These authors identified more than 90% of genomic regions underlying selection signatures had European taurine origin. Vahedi et al. explored three haplotype-based methods (iHS, iHH12, and nSL) for selection signatures aiming to identify more recent systematic artificial selection following the breed formation than old selective sweeps. Interestingly, most of the selection signatures and indicine-taurine differentiated genomic regions were breed specific in both studies, suggesting that differences in breeding objectives and selection intensities exist between the composite breeds. The only exception in most of the recent studies with composite cattle breeds is found in chromosome 5, which consistently had a high indicine ancestry (Paim et al., 2020; Crum et al.; Mulim et al.; Vahedi et al.). This warrants further exploration to elucidate the high indicine ancestry in chromosome 5. These papers demonstrated how complementarity and selection jointly contribute to shape the genetic architecture of the Composite breeds population.

Mulim et al. characterized a Brazilian composite beef cattle breed known as Purunã, which was formed by crossing Angus, Charolais, Canchim, and Caracu. Runs of homozygosity (ROH) analyses showed low inbreeding levels with low correlations with pedigree-based measures. These authors identified heterozygote islands harboring genes involved in growth pathways, carcass weight, meat and carcass quality, and marbling deposition. This four-breed composite population had low consistency of gametic phase with the founder breeds, therefore multi-breed genomic evaluation is likely not feasible (Mulim et al.). Composite breeds formed by more than two breeds and with multiple taurine and indicine founders can have a different genetic architecture than previously studied two-breed composite populations such as the Montana Tropical beef cattle (Grigoletto et al., 2020).

There are numerous local breeds in Asia, in which various of them were included in this Research Topic. Most of the Chinese cattle breeds have a complex breeding history and migration, with different combinations of Taurine (European and Asian) and Indicine (Chinese and Indian) origin (Freitas et al., 2021). These papers presented genome sequencing of different Chinese breeds, as Weining (Liu et al.), Dianzhong (Zhang et al.), Lincang Humped Cattle (Sun et al.), Dengchuan (Jin et al.); Xiangxi (Luo et al.). All of them identified genomic selection signatures related to environmental adaptation, such as cold adaptation associated with fat metabolism and blood pressure regulation (Liu et al.), adaptation to hot and humid climate (Zhang et al.), and body size, immunity, and heat tolerance (Sun et al.). These authors also reported missense mutations in the *HELB* gene that were specific to indicine cattle and were presumed to be associated with adaptation to hot environments. These studies provide new insights into the genetic background of Chinese cattle populations, which represent an important reservoir of cattle genetic diversity for future uses, especially considering the emerging challenges imposed by climate change. For instance, the Dengchuan cattle is the main local yellow dairy cattle breed in China with high milk fat percentage and a local specialty dairy product. This is an endangered population among Yunnan native cattle breeds, threatened mainly by crossing with the exotic Holstein breed (Jin et al.). Moreover, these authors showed that Yunnan has been one of the core regions for the migration of Indian indicine into the Chinese territory.

Chinese composite pig breeds (Xidu and Beijing black pig) were also studied as part of this Research Topic. Xu et al. reported genes within ROH islands related to reproduction, fat deposition, ear shape, and environmental adaptation in Xidu black pigs. Population genetic differentiation (F_ST_) of Beijing Black pigs and other populations ranged from 0.10 to 0.27, which showed that Beijing Black pigs were more genetically similar to the commercial pig breeds than Chinese local pigs, retaining a small amount of Huainan and Min pigs (Yang et al.). This study provides new insights into the historical contribution of Western and Chinese ancestry to actual Beijing Black pigs.

Korean synthetic pig breed (Woori-Heukdon—Korean native pig x Duroc) had more stable genomic breed composition in the first generations. Short ROH reduced while medium and long ROH increased from F1 to WRH, suggesting that more recent inbreeding is happening at a higher rate in WRH (Kim et al.). Therefore, the authors indicated the need for better inbreeding management in these composite breeds.

Guo et al. studied Yunnan semi-fine wool sheep based on whole-genome resequencing data, using Tajima´s D, iHS, and fisher test (comparing groups of one or two lambs per gestation). These authors identified genomic regions associated with environmental adaptation (cold climate, high altitude, and hypoxic conditions) and litter size.

Dong et al. sequenced one Mongolian Kulan and 29 Kulan hybrids. These authors identified the important contribution of the *KITLG* gene to coat color. Mongolian Kulan is an essential part of Asiatic wild ass, but hunting and deteriorating living conditions have caused their numbers to plummet leading them to nearly the level of a threatened species in the International Union for Conservation of Nature Red list (Dong et al.).

Chen et al. studied copy number variation (CNV) in six chicken breeds (four Chinese indigenous breeds and two commercial breeds), which provided an interesting perspective on the evolutionary spectrum of CNVs under artificial selection during chicken domestication and breed formation. Important candidate genes contributing to fast growth, high reproduction, and distinct breed characteristics were identified by Chen et al. This study is a valuable resource to facilitate genetic and functional investigation of domestication and economic traits in chickens.

Qiao et al. provided the first hair sheep reference genome, representing a valuable resource for sheep genetic studies, and provided a pipeline for mining genetic information of composite breeds based on detection of allele-specific expression genes. According to these authors, Dorset sheep had a greater impact in the growth rate, carcass quality, and carcass yield of Dorper sheep than the Persian breed. The Persian breed seems to have contributed more to traits related to fat deposition.

Overall, this Research Topic is a first step towards better characterizing breed formation, genetic architecture, and selection signatures in composite livestock populations. Moreover, many new research insights may arise from the results and discussion presented in the studies included in this Research Topic.
